# Helmholtz Versus Haute Couture: How Horizontal Stripes and Dark Clothes Make You Look Thinner

**DOI:** 10.1177/03010066211038158

**Published:** 2021-08-16

**Authors:** Antonis Koutsoumpis, Elias Economou, Erik van der Burg

**Affiliations:** 1190 Vrije Universiteit Amsterdam, Netherlands; University of Crete, Greece; Netherlands Organization for Applied Scientific Research, 2859 TNO, Netherlands; 1234 University of Amsterdam, Netherlands

**Keywords:** size perception, horizontal stripes, luminance, Helmholtz illusion, clothes

## Abstract

In Helmholtz’s illusion, a square with horizontal stripes appears taller than an identical square with vertical stripes. This effect has also been observed in experiments with human stimuli, where a human figure wearing a dress with horizontal stripes appears thinner than a drawing clad in vertical stripes. These findings do not agree with the common belief that clothes with horizontal stripes make someone appear wider, neither do they disentangle whether the horizontal or vertical stripes account for the thinning effect. In the present study, we focused on the effect of horizontal stripes in clothes comparing horizontal stripes against no-stripes (not against vertical; Experiments 1 and 2), using photos of a real-life female model, and controlling for the average luminance of the stripes (Experiment 2). Results showed that horizontal stripes and lower luminance have—independently—a small-to-moderate thinning effect on the perceived size of the body, and the effect is larger when the two variables are combined. In Experiment 3, we further show that the thinning effect due to the luminance of the dress is enhanced when the general background gets darker.

Do clothes with horizontal stripes make us look fatter? The common belief is that they do. Interestingly, not only horizontal stripes are frequently quite fashionable, but empirical evidence also supports the opposite: horizontal stripes seem to have a thinning effect on the perceived body width. The first to report a thinning effect of horizontal stripes was Helmholtz (1867/1962) who showed that a square filled with horizontal stripes was perceived as taller (and therefore thinner) than an identical square filled with vertical stripes, an effect known as the *Helmholtz illusion*. Thompson and Mikellidou (2011) quantified this illusion reporting that a square with horizontal stripes needs to be extended 4.5% to match the width of a similar square with vertical stripes.

A number of studies have investigated whether the Helmholtz illusion generalizes to clothes. For instance, Thompson and Mikellidou (2011) showed participants the line drawing of two female figures wearing a dress. One dress had vertical and the other horizontal stripes. The width of the female figure wearing the horizontal stripes was systematically manipulated, and participants were instructed to judge which of the two figures was wider (the one with the vertical or the one with the horizontal stripes). Thompson and Mikellidou found that the stimulus with the horizontal stripes had to be 5.8% broader than the one with the vertical stripes to be perceived as equal in size, verifying Helmholtz illusion using different stimuli. Similar results were obtained when Thompson and Mikellidou (2011) asked participants to view the photographs of two female mannequins wearing strapless t-shirts with vertical or horizontal stripes stereoscopically. The horizontally striped mannequin had to be 10.7% wider to be perceived identical to the vertically striped one, indicating that the thinning effect of horizontal stripes generalizes to 3D stimuli.

In a more recent study, Ashida et al. (2013) examined whether the thinning effect of horizontal stripes depends on body size. In their study, participants saw the image of a female model wearing a shirt with either horizontal or vertical stripes and asked participants to match the size of the vertically striped stimulus with thinner and wider versions of the horizontally striped stimulus. They also manipulated the body size of the model (creating a thinner and an oversized version of the figures) to test whether the effect persists across different body sizes. Their results replicated the Helmholtz illusion for both body sizes, with the thinning effect being stronger for the thinner than the oversized body.

However, not all research findings on Helmholtz illusion are unequivocal. For example, Imai (1982; as reported by Ashida et al., 2013; original paper in Japanese) using the drawing of a—rather overweight—male figure wearing either a horizontally or vertically striped shirt, found that the vertically striped stimulus was judged 15.37% thinner than that with horizontal stripes. These findings suggest that for larger body sizes horizontal stripes have a widening effect, opposite to the Helmholtz's illusion.

The studies reported so far have tested the thinning effect of horizontal stripes by directly comparing it against vertical stripes. However, the direct comparison between horizontal versus vertical stripes fails to inform us which stripe orientation produces the effect, leaving it unclear whether clothes with horizontal stripes make someone look thinner, or whether clothes with vertical stripes make someone look wider, or potentially both. And, while it is true that the most common approach in illusion studies is to report the overall effect, it might be useful to know what each element of the illusion is contributing, especially if one wants to model the illusion. This has rarely been done with size illusions, but it has found application in the explanation of lightness illusions (Economou et al., 2007). To examine whether wearing horizontal stripes make someone look thinner, one should de-anchor horizontal stripes from vertical, testing their effect either in isolation or compared to a non-striped stimulus (i.e., a neutral condition).

This direct comparison between stimuli with stripes (either horizontal or vertical) and non-stripes was tested by Swami and Harris (2012) in a naturalistic setting. In their study, participants interacted with a real female confederate dressed in a striped (horizontally or vertically) or non-striped dress, in random order. Shortly after a brief interaction, participants were asked to assess the size of the confederate using an objective index of female body size (Fotographic Figure Rating Scale; Swami et al., 2008). Interestingly, and contrary to the Helmholtz illusion, a significant widening effect of horizontal stripes was observed, both as compared to vertical (Cohen's *d* = 0.63) and to non-stripes (Cohen's *d* = 0.61), corroborating the common belief that horizontal stripes make someone look wider. The study by Swami and Harris added ecological validity to the effect, since it resembled the judgment process followed in natural surroundings (e.g., when deciding to buy a new piece of clothing). However, the experimental method lacked a clear focus on the perceptual nature of the task and rather introduced confounding variables, since participants were asked to assess from memory a stimulus they were not instructed to pay attention to. It is well known that people are poor at perceiving unattended stimuli in their environment (see e.g., the “invisible gorilla” experiment on inattentional blindness; Simons & Chabris, 1999), and that perception accuracy diminishes as the interval between the test and response phase increases (Bowen et al., 2016; Bradshaw & Watt, 2002). Therefore, it is unclear whether the task assessed a perceptual process or whether participants responded based on their previous beliefs (i.e., a response bias).

The comparison between horizontally and non-striped stimuli was tested in a laboratory setting by Thompson and Mikellidou (2011; Experiment 3), employing not a human figure but a geometrical shape as stimulus. They asked participants to estimate the width of a cylinder with horizontal, vertical, or no stripes (colored in uniform gray), by adjusting the distance of two vertical lines. When compared against the neutral, non-striped condition, the thinning effect of horizontal stripes disappeared (on the other hand, the vertically striped cylinder was perceived significantly wider than non-striped cylinder), suggesting that the Helmholtz illusion emerges only when horizontal stripes are compared to vertical, but not when compared to a non-striped stimulus.

To test whether this also applies to clothes, and since no previous study has tested whether a human figure wearing a garment with horizontal stripes results in a thinning effect compared to an object without stripes (i.e., a neutral condition), we compared the perceived width of a female model wearing a dress with horizontal stripes against a similar stimulus without stripes. Following Thompson and Mikellidou’s (2011; Experiment 3) findings, we hypothesized that a dress with horizontal stripes will have no thinning effect when compared against a similar dress with no stripes.

As compared to previous experiments, the present study differentiates in two ways. First, the stimuli used in previous studies were either line drawings of human figures (Imai, 1982; Thompson & Mikellidou, 2011) or computerized images (Ashida et al., 2013; with the exception of the stereoscopically seen mannequins in Thompson & Mikellidou, 2011, Experiment 4), not necessarily close to what a real human figure looks like. To improve stimuli's realism, we used the photo of a real female model wearing a dress with horizontal stripes (green-black) scraped from the internet. After editing the image (The GIMP Development Team, 2019), a green and a black non-striped version of the model were created. The face and legs of the model were removed to avoid attractiveness effects. Figure 1 displays the original striped and the two edited non-striped versions of the dress.

**Figure 1.**
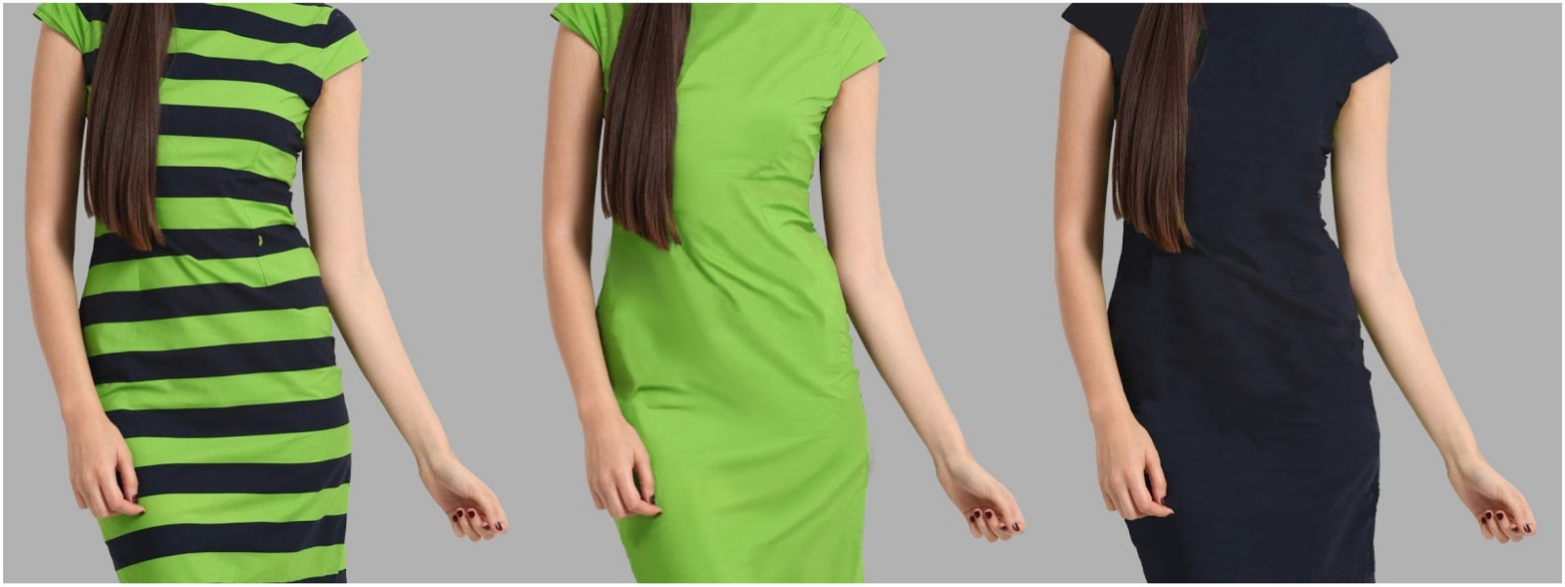
Stimuli used in Experiment 1. From the original green-black striped version (left), two non-striped versions were created (green and black).

Second, previous studies instructed participants to judge which of the two stimuli looked fatter/thinner. Typically, such a response results in S-shaped distribution and the mean of the distribution corresponds to the point of subjective equality (PSE; i.e., the point at which the two stimuli are perceived equally wide). Although the PSE in previous studies may reflect a perceptual effect, in some cases it might reflect a response bias, since participants may consistently prefer choosing one stimulus over another (see e.g., Alais et al., 2014; Van der Burg et al., 2008; Van Eijk et al., 2008, for a similar logic). Therefore, in the present study, we decided to apply the method of adjustment (i.e., adjust one of two stimuli until they are perceived similar), so that participants were unable (or at least find it more difficult) to directly favor one stimulus over the other. To facilitate readability, since this paper is only concerned with the perceptual effect of horizontal stripes, throughout the manuscript the word “stripes” will refer specifically to horizontal stripes, except where otherwise specified.

## Experiment 1

**Figure 2.**
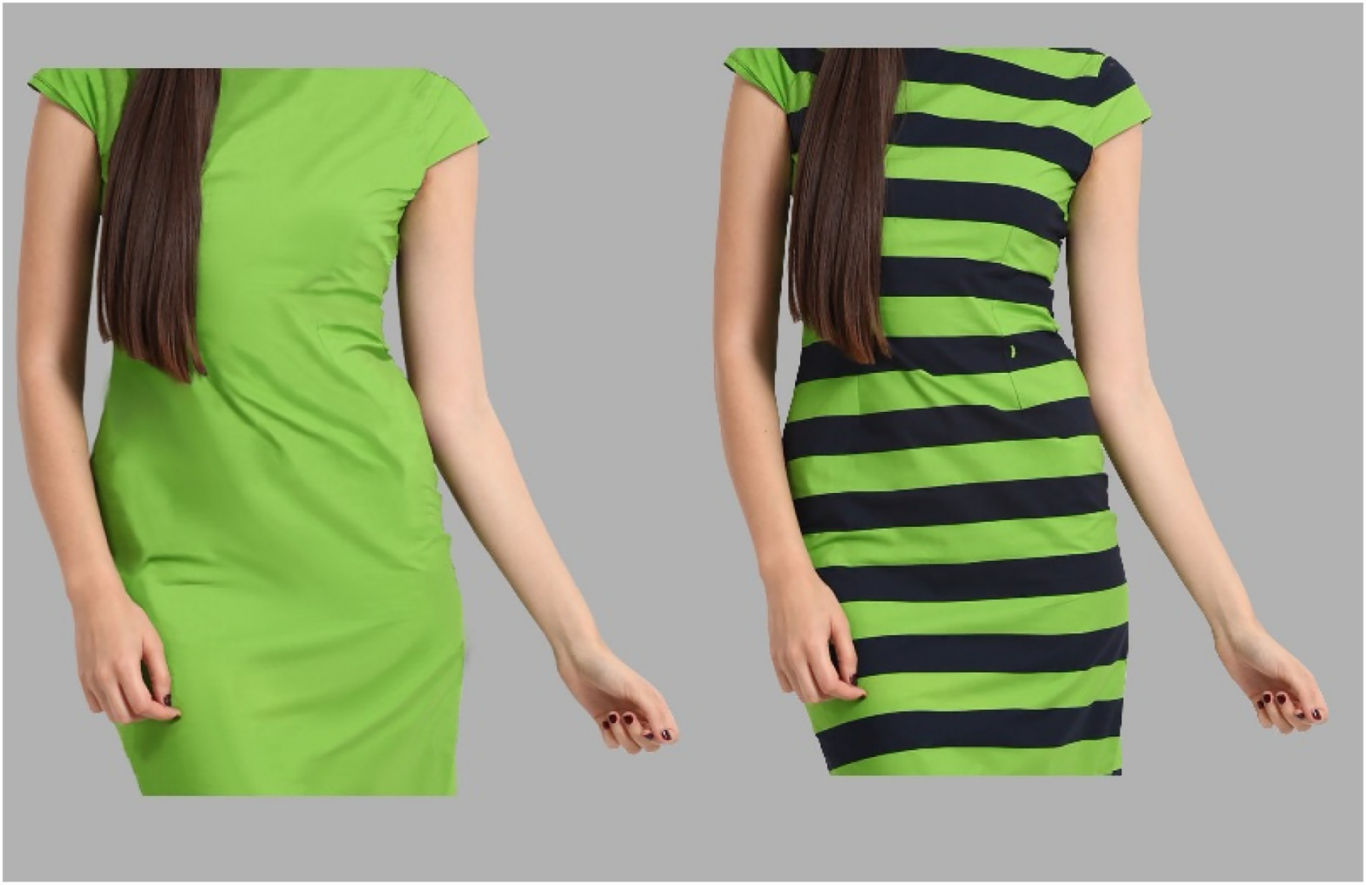
Example display used in Experiment 1. On each trial, participants saw a female body wearing a dress with stripes, and another without stripes. The width of one body (the standard) was fixed (here the one with the stripes), whereas the width of the other body (the variable) was randomly determined. Participants were instructed to adjust the width of the variable body until they perceived both bodies as equally wide. A word cue informed participants which body to adjust.

The aim of Experiment 1 was to examine whether horizontal stripes in clothes—compared to non-stripes—have a thinning effect on the model's appearance. On every trial, participants saw two stimuli on the monitor: a female model wearing a dress with horizontal stripes (green/black with a 50% duty cycle), and another one wearing the same dress without stripes (either green, or black). The width of one model (the standard) was fixed, whereas the width of the other (the variable) was randomly determined. An example display is illustrated in Figure 2. Participants were instructed to adjust the width of the variable stimulus until they perceived both bodies as equally wide (i.e., the point of subjective equality; PSE). Note that a PSE of zero indicates that both stimuli are perceived as equally wide. Before each trial, a word cue informed participants which body to adjust (either the striped or non-striped one). If horizontal stripes make a body look thinner, we would expect the PSE to be significantly larger than zero when the variable stimulus is the striped dress. We expected the opposite effect when the variable stimulus is the non-striped dress.

## Method

### Participants

Forty-three participants (10 males and 33 females; mean age was 20.6 years old, *SD* = 3.8 years, ranging from 18 to 35 years) took part in Experiment 1, which lasted approximately 10 min. All participants were naïve as to the purpose of the experiment and were paid €4 or received course credits for their participation. All participants provided written consent. The experiment was approved by the local ethics committee of the Vrije Universiteit Amsterdam and conducted in accordance with the Declaration of Helsinki.

### Stimuli and Apparatus

The experiment was programmed in python using the OpenSesame software (Mathôt et al., 2012). Participants were seated in a dimly lit cubicle at a distance of approximately 72 cm from the 22 in. monitor (Samsung SyncMaster; refresh rate 120 Hz, resolution 1,680 × 1,050 pixels). A standard QWERTY keyboard was used for monitoring responses.

On every trial, participants saw a female body wearing a dress with stripes, and another one wearing a dress without stripes on a general light gray background (luminance 49 cd/m^2^). Figure 2 illustrates an example display used in Experiment 1. The non-striped dress was either light-green (39 cd/m^2^; in all stimuli we measured the average luminance in the belly-waist area), or black (<0.5 cd/m^2^), whereas the striped dress was a combination of both colors (green and black horizontal stripes; the width of a single stripe was approximately 0.98°, and the duty cycle was set to 50%; luminance was 2 cd/m^2^). The height of both images was fixed (18.75° visual angle), whereas the width of the bodies varied between 13.20° to 18.41° (width measured as the maximum distance between stimulus’ left-right hand), in steps of 0.087°. From the two images on the screen, the width of one body (the standard) was fixed (15.81°), whereas the initial width of the other body (the variable) was randomly determined (±1.73°) in order to avoid a hysteresis effect. The center of the images was located on the left and the right from the center of the screen (distance was 9.11°), and the *x* and *y* coordinates were jittered (±0.43°). The body wearing the striped dress was randomly presented on the left or the right side of the screen, with equal probabilities to appear on either side.

### Procedure and Design

At the beginning of each trial a word cue, presented for 500 ms, informed participants about the stimulus they had to adjust (variable). The cue was either the word “stripes” when participants were asked to adjust the width of the body wearing the striped dress, and “green” or “black” when participants were instructed to adjust the width of the body wearing the non-striped dress. Subsequently, the two bodies were shown and participants were instructed to adjust the width of the variable body until they perceived both bodies to be equally wide (i.e., the PSE). Participants pressed either the upper or lower arrow key to increase or decrease the body width, respectively, and the spacebar to submit their response once they were satisfied. There was no time limit in their responses. The dependent variable was the mean PSE. The independent variables were the type of stripes on the dress (stripes or non-stripes), and the color of the non-striped dress (black or green). Each condition was counter balanced and the order was randomly determined. Each combination was repeated 15 times, making a total of 60 trials. Participants received instructions on the screen prior to the experiment and practiced a couple of trials to familiarize themselves with the task.

## Results

**Figure 3.**
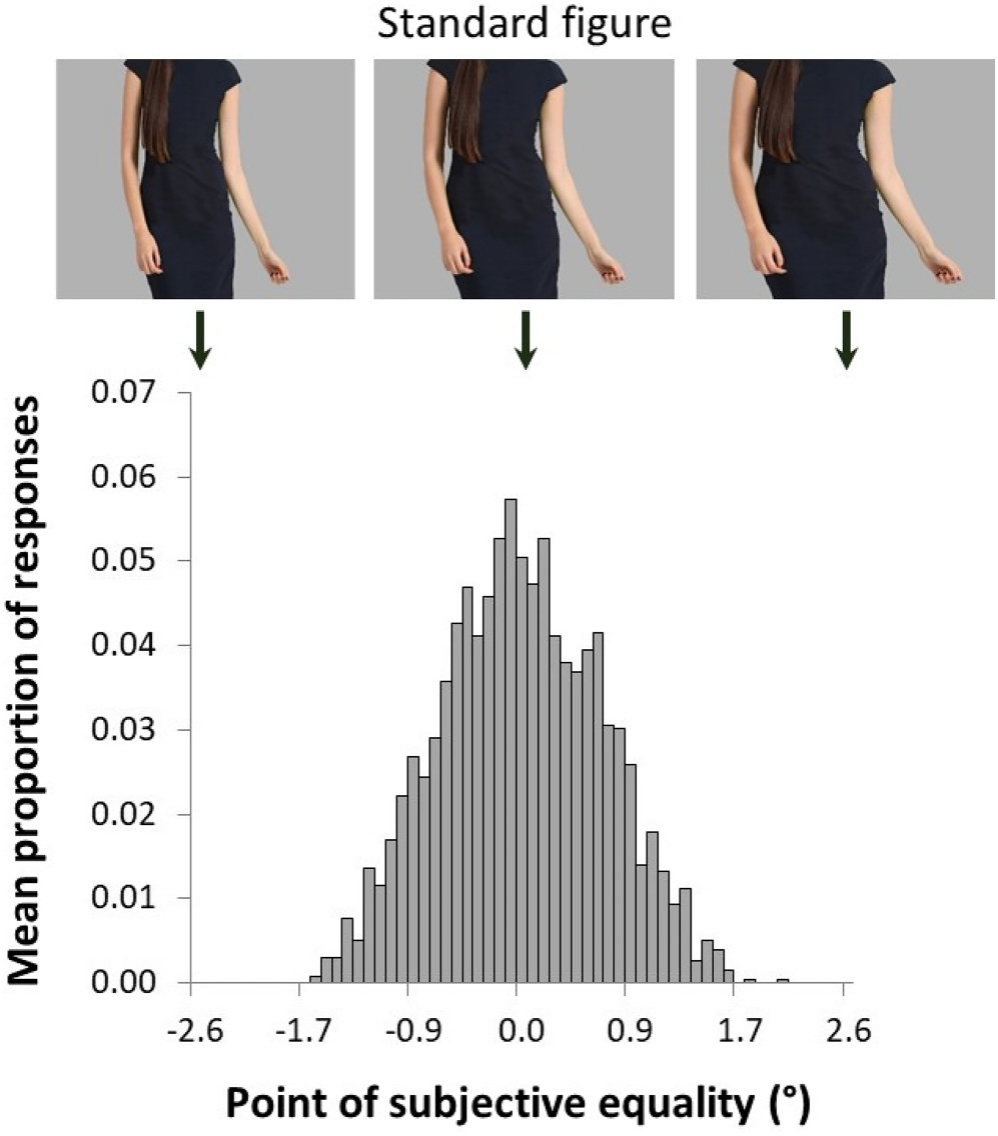
Distribution of responses per image, representing the point of subjective equality (PSE). The figure above the distribution represents the boundary conditions (left and right female figures) and the standard female figure (center). Note that this distribution is not specific for the figure wearing the black dress, as we collapsed over all conditions.

For illustrative purposes, the distribution of responses per image is shown in Figure 3. Here the group mean proportion of responses is shown for each image (representing the PSE) collapsed over all conditions. Figure 3 shows the distribution of responses around the standard image (PSE = 0°).^1^

### Mean Point of Subjective Equality

**Figure 4.**
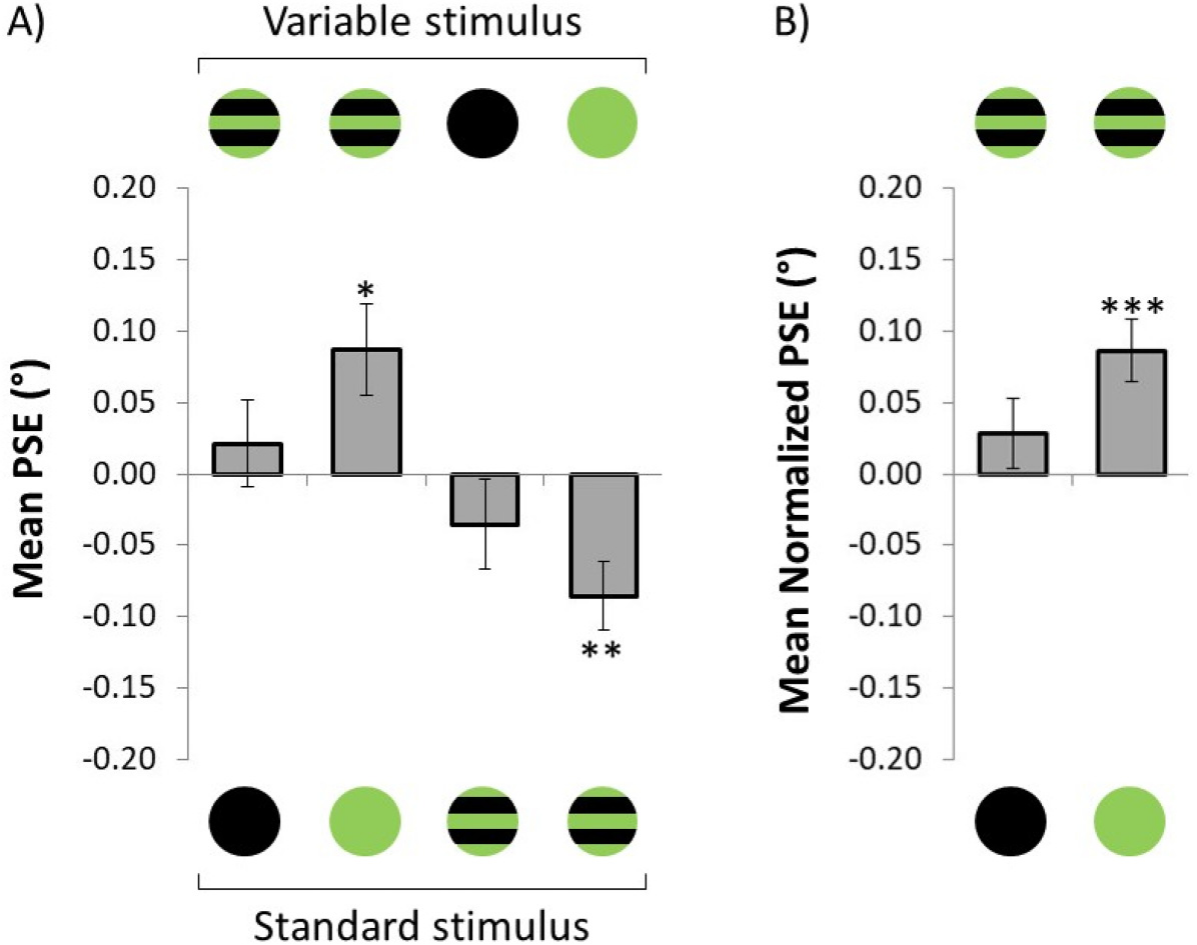
Results of Experiment 1. (A) Mean PSE for each dress type of the variable body (upper part) and the dress of the standard body (lower part). (B) Normalized mean PSE as a function of the color of the dress without stripes. A positive normalized PSE signifies that the dress above the *x*-axis is perceived as thinner than the dress below the *x*-axis, and vice versa. The error bars represent the *SEM*. *p < .05, ** *p* < .01, *** *p* < .001.

Abbreviations: PSE = point of subjective equality; *SEM* = standard error of the mean.

The results of Experiment 1 are shown in Figure 4A. Here, the mean PSE is plotted for each dress type of the variable body (upper part of the graph) and the standard body (lower part). Since multiple *t*-tests were conducted, to avoid family-wise error rate, all *p*-values across the manuscript have been adjusted following the Bonferroni-Holm method (Holm, 1979).

In the comparison between the striped and non-striped light-green dress, the mean PSE was significantly larger than zero when the variable body was wearing a dress with stripes (PSE = 0.09°, *t*_42_ = 2.75, *p* = .03, *d* = 0.42, 1 − *β* = 0.77),^2^ and the mean PSE was significantly smaller than zero when the variable body was wearing a dress without stripes (PSE = −0.09°, *t*_42_ = −3.55, *p* = .004, *d* = 0.54, 1 − *β* = 0.93). When the striped dress was compared to the black dress, no significant difference was observed neither when the variable body was the striped (PSE = 0.02°, *t*_42_ = 0.69, *p* = .53, *d* = 0.11, 1 − *β* = 0.10) nor when it was the black dress (PSE = −0.04°, *t*_42_ = −1.13, *p* = .53, *d* = 0.17, 1 − *β* = 0.20).

### Mean Normalized Point of Subjective Equality

Provided that we are not interested in whether participants were adjusting the body wearing stripes or the one without stripes, as this will simply result in opposing effects (see Figure 4A), we decided to normalize the PSE using equation (1).

(1) $$\mathrm{normalized}\;\mathrm{PSE}(x,y)=\frac{\mathrm{PSE}\;(\mathrm{adjust}\;x)-\mathrm{PSE}(\mathrm{adjust}\;y)}{2}.$$

Here, the normalized PSE reflects the difference between the PSE when body *x* is the variable compared to when body *y* is the variable. A positive normalized PSE value reflects that body *x* is perceived as thinner than body *y*, and vice versa. Figure 4B illustrates the mean normalized PSE as a function of the color of the dress without stripes. Here, and elsewhere in the manuscript, the dress above the graph reflects body *x*, whereas the dress below the graph reflects body *y*. According to this definition, each effect (i.e., the sign) points toward the dress that is perceived as thinner. As revealed by a two-tailed *t*-test, the striped dress was perceived as thinner than the non-striped light-green dress (PSE = 0.09°, *t*_42_ = 3.98, *p* < .001, *d* = 0.61, 1 − *β* = 0.97). However, the body wearing stripes was not significantly different than the body wearing a black dress (PSE = 0.03°, *t*_42_ = 1.13, *p* = .26, *d* = 0.17, 1 − *β* = 0.20).

The results suggest that the striped dress was perceived as thinner than the non-striped dress. However, this effect was predominantly observed when the non-striped dress was light-green, but not when it was black. A likely explanation for this contrasting effect might be the luminance of the non-striped dresses. According to the irradiation illusion (Helmholtz, 1962), bright stimuli on a dark surface look larger than similar in size dark stimuli on a bright surface (Long & Murtagh, 1984; Oyama & Nanri, 1960). As a result, it might be feasible that the dark dress (low luminance) makes a body look thinner than a body wearing a bright dress (high luminance), suggesting that some of the thinning effect might be explained by the luminance difference between the stimuli.

The difference between the striped and non-striped green dress could also be attributed to luminance, since by removing the—black—stripes, the luminance of the non-striped dress increases. If the “horizontal” and “luminance” effects combine (i.e., additive), this would explain why the striped dress looked thinner when compared to the non-striped light-green dress, but not when compared to the black dress, since the luminance effect is in the opposite direction of the stripes effect. To disentangle between the potential stripe and luminance effects, we conducted Experiment 2.

## Experiment 2

The aim of Experiment 2 was twofold. First, we aimed to replicate the findings of Experiment 1. Second, we aimed to further explore the nature of the illusion by decomposing it into two separate effects: the potential effect of horizontal stripes controlling for any effect of luminance, and the potential effect of luminance controlling for the effect of horizontal stripes. The procedure was identical to Experiment 1, but we introduced two novel stimuli to control for the effect of stripes and luminance. We expected that a body clad in a striped dress would be perceived as thinner than a body clad in a non-striped dress when both stimuli have the same luminance (stripes effect). Furthermore, we expected that the luminance of a dress plays a crucial role in the perception of the body width, such that darker stimuli will be perceived as thinner, regardless of whether those dresses have stripes or not (luminance effect).

## Method

### Participants

Forty-five participants (due to a mistake in demographic data collection, data of only 31 participants were stored: 5 male, 26 female; mean age was 20.77 years old, *SD* = 3.93 years, ranging from 18 to 39 years) took park in Experiment 2. The experiment lasted approximately 40 min. All participants were naïve as to the purpose of the experiment and were paid €7 or received course credits for their participation.

### Stimuli and Apparatus

**Figure 5.**
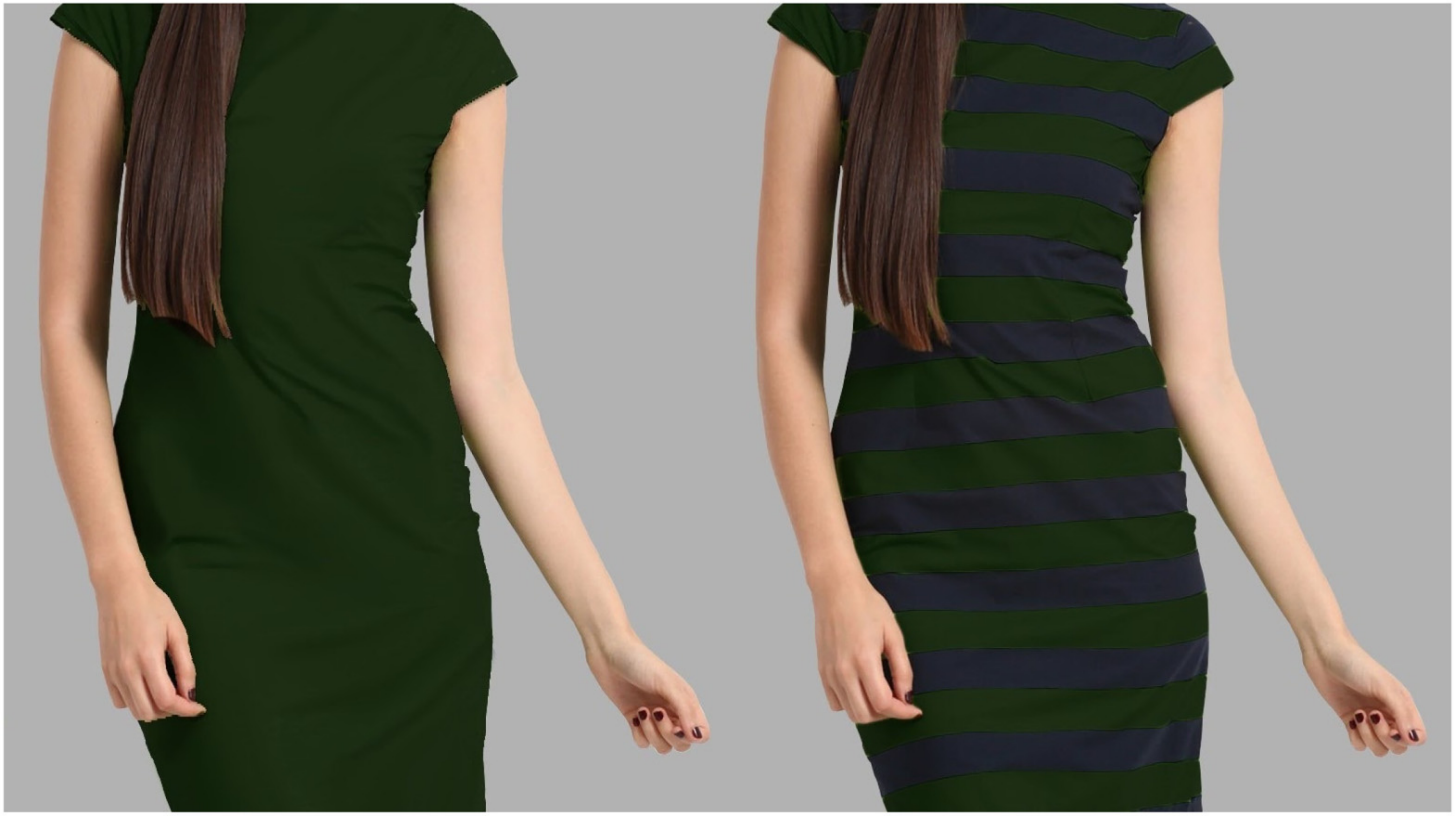
The non-striped- (left) and striped- (right) dark-green dresses used in Experiment 2.

The experiment was identical to Experiment 1, with two exceptions. First, in contrast to Experiment 1, we used five different dresses as stimuli (two new ones in addition to the three used in Experiment 1). The two new dresses were a non-striped dark-green dress with luminance 2 cd/m^2^ (the luminance is deliberately identical to that of the light-green striped dress of the previous experiment), and the striped version of the same dark-green dress (average luminance = 1 cd/m^2^; see Figure 5). Second, unlike Experiment 1, we decided not to apply a full factorial design since we were not interested in all possible combinations, and since a full factorial design would lead to an unnecessarily long-lasting experiment.

The inclusion of the extra stimuli allowed to test the effect of one variable (e.g., horizontal stripes/luminance) when controlling for the effect of the other, but also to test the aggregating effect of both variables. More specifically, the experimental conditions allowed to test for three effects. First, the effect of horizontal stripes controlling for the effect of luminance was tested in one condition: *dark-green versus stripes light-green* (the two dresses have the same luminance). Second, the luminance effect controlling for the effect of stripes was tested across two conditions: *stripes light-green versus stripes dark-green* and *light-green versus dark-green* dress. Third, to test whether the effects of stripes and luminance sum together we used five conditions: *dark-green versus stripes dark-green*, *light-green versus stripes dark-green*, *black versus stripes light-green*, *light-green versus stripes light-green*, and *black versus stripes dark-green*. As in Experiment 1, each dress served as the variable stimulus on 50% of the trials and as the standard on the remaining trials. The word cue was either “stripes dark-green” or “stripes light-green” when participants were asked to adjust the width of the body wearing the dress with stripes, and “dark-green,” “light-green,” or “black” when participants were asked to adjust the width of the body wearing the dress without stripes. In total, there were 240 trials (15 trials ×  2 [variable, or standard] × 8 conditions). The order of the trials was randomized for all participants.

## Results

**Figure 6.**
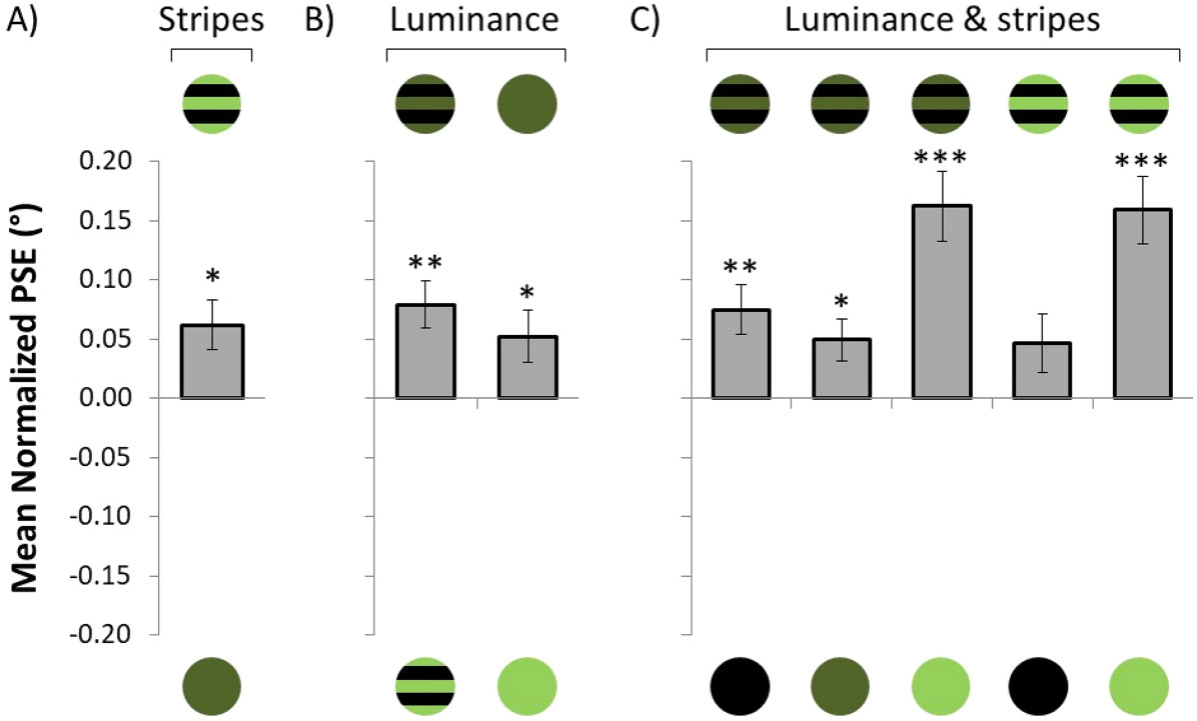
Results of Experiment 2. Here, the mean normalized PSE is plotted for each type of the dresses used. (A) Stripes effect on the perceived body width (controlling for luminance effect), as both dresses have the same luminance. (B) Luminance effect (controlling for stripes effect) on the perceived body with, as both dresses were either striped or both non-striped. (C) Aggregate luminance and stripes effect on the perceived body width. A positive normalized PSE signifies that the dress above the *x*-axis is perceived as thinner than the dress below the *x*-axis, and vice versa. The error bars represent *SEM*. * *p* < .05, ** *p* < .01, *** *p* < .001.

Abbreviations: PSE = point of subjective equality; SEM = standard error of the mean.

Figure 6 illustrates the mean normalized PSE for each dress type of the variable body (upper part of the graph) and the standard body (lower part) for the stripes, luminance, and combined effects.

### Stripes Effect

The effect of stripes is shown in Figure 6A. The striped light-green dress was perceived as thinner than the non-striped dark-green dress (PSE = 0.06°, *t*_44_ = 2.50, *p* = .049, *d* = 0.37, 1 − *β* = 0.69). Thus, when the effect of luminance was controlled for (the comparison included only stimuli of same luminance), striped stimuli were perceived as thinner compared to non-striped stimuli.

### Luminance Effect

The effect of target luminance is shown in Figure 6B. Two-tailed *t*-tests were conducted on the PSE. Stimuli with lower luminance were perceived as thinner, both for striped (PSE = 0.08°, *t*_44_ = 3.90, *p* = .002, *d* = 0.58, 1 − *β* = 0.97) and non-striped dresses (PSE = 0.05°, *t*_44_ = 2.37, *p* = .049, *d* = 0.35, 1 − *β* = 0.64). Thus, when the effect of stripes was controlled for, dresses with lower luminance were perceived as thinner.

### Luminance and Stripes Effect

The combined effect of luminance and stripes is shown in Figure 6C. The dress with dark-green stripes was perceived thinner when compared to black (PSE = 0.07°, *t*_44_ = 3.64, *p* = .004, *d* = 0.54, 1 − *β* = 0.94), non-striped dark-green (PSE = 0.05°, *t*_44_ = −2.80, *p* = .03, *d* = 0.42, 1 − *β* = 0.78) and light-green (PSE = 0.16°, *t*_44_ = 5.54, *p* < .001, *d* = 0.83, 1 − *β* = 0.99). The dress with light-green stripes was perceived as thinner compared to non-striped light-green (PSE = 0.16°, *t*_44_ = 5.60, *p* < .001, *d* = 0.83, 1 − *β* = 0.99) and marginally significantly thinner when compared to black (PSE = 0.05°, *t*_44_ = 1.88, *p* = .067, *d* = 0.28, 1 − *β* = 0.47). These results replicate the findings of Experiment 1.

Although our design did not allow for a direct test of the interaction between the two main factors (stripes, luminance), the data seem to indicate that when stripes and low luminance are taken together, they produce an even larger thinning effect. Bars 3 and 5 (Figure 6C) illustrate this point. In those conditions the variable stimuli have both lower luminance and contain stripes compared to the standard stimuli, and are the conditions where the largest thinning effect is observed. One might expect an even larger thinning effect for bar 3 since—although both striped stimuli in bars 3 and 5 are compared against the same light-green dress—the luminance of the variable stimulus in bar 3 is lower than the one in bar 5. However, we think that the absence of a difference between these conditions probably reflects a ceiling effect. The only comparison contrary to the hypothesis that the independent effects of stripes and low luminance seem to combine and produce an even larger thinning effect, is the one between bars 1 and 2 (Figure 6C). One would expect a larger thinning effect for bar 2 compared to bar 1, since, although the variable stimuli are the same, the standard stimulus in bar 1 has lower luminance than the one in bar 2—thus “antagonizing” the thinning effect of the stripes and luminance more strongly. Note however, that the luminance difference between the variable and standard stimuli in bars 1 and 2 is no more than 1 cd/m^2^, meaning that in these two conditions the effect of luminance contributes very little to the overall effect, and the PSE difference is rather attributable to the thinning effect of stripes rather than that of luminance.

Although the results seem to support the notion that both stripes and dress luminance have a thinning effect, one might argue that dress luminance is confounded with background luminance, since across all conditions two relatively dark stimuli (variable and standard) are placed on a relatively bright background (i.e., all stimuli have lower luminance than the background). Consequently, the thinning effect could theoretically be the result of the higher contrast produced between dark dresses and the light-grey background, and not of the low luminance of the dress itself. To test whether figure-background contrast affected the perceived thinning effect, we conducted Experiment 3.

## Experiment 3

The role of the contrast between the dress and its background in the thinning effect was not explicitly studied in Experiments 1 and 2. It could be argued that the higher the contrast, the stronger the thinning effect that was observed across conditions. However, this would be difficult to justify in the case of the striped dress condition. In the stripes effect (Figure 6A), the two stimuli had the same luminance, thus they were equally distant from the background luminance. At the same time, the results showed that, when the luminance of the stimuli and the background luminance were held constant, horizontal stripes seemed to account for the thinning effect, regardless of any stimuli or background luminance effect. Therefore, it would be highly unlikely that the thinning effect produced by the stripes was accounted for by the contrast between the dresses and their backgrounds. However, regarding the luminance effect (Figure 6B), although darker stimuli produced a thinning effect, either when they were striped (bar 1) or non-striped (bar 2), the luminance of the constant and variable dresses was different. Therefore, the thinning effect could be attributed to the luminance difference between the two dresses, but it could also have been affected by the different contrast between the dresses and the background, since the darker stimuli (which were perceived as thinner) contrasted more with their backgrounds as compared to the brighter stimuli.

In Experiment 3, we kept the luminance of the dark and bright dresses constant and manipulated the background luminance to test whether it affected size perception. We decided to replicate the findings of Figure 6B, focusing on the non-striped dresses (bar 2), since the luminance difference between the stimuli was larger than those in bar 1. We tested three grey backgrounds with different levels of luminance: bright (similar luminance to Experiments 1 and 2; background luminance was 1.26% brighter than the brightest dress), average (average luminance between the two dresses), and dark (background luminance was 1.26% darker than the darkest dress). If contrast was the driving force behind the thinning effect for low luminance dresses in Experiment 2, we would expect that as this contrast was reduced (i.e., the background gets darker and darker) the thinning effect would be also reduced.

## Method

### Participants

Twenty participants (16 women; mean age was 24.45 years old, *SD* = 4.59 years, ranging from 18 to 35 years) took park in Experiment 3. The experiment lasted approximately 15 min. All participants were naïve as to the purpose of the experiment and received course credits for their participation. One participant was excluded from analyses, as he or she selected the first random variable dress (without any adjustment of the stimulus) as being equal to the standard dress on 35% of the trials (compared to the group mean: 1.9%).

### Stimuli and Apparatus

The experiment was identical to Experiment 2, with two exceptions. First, in contrast to Experiment 2, we used only the dark-green (2 cd/m^2^) and the light-green (39 cd/m^2^) dress (both without stripes). Second, we manipulated the background luminance, creating dark- (1.5 cd/m^2^), average- (20.5 cd/m^2^) and bright-grey (49 cd/m^2^; similar to Experiments 1 and 2) backgrounds. In total, there were 90 trials (15 trials × 2 [variable, or standard] × 3 conditions). The order of the trials was randomized for all participants.

## Results

**Figure 7.**
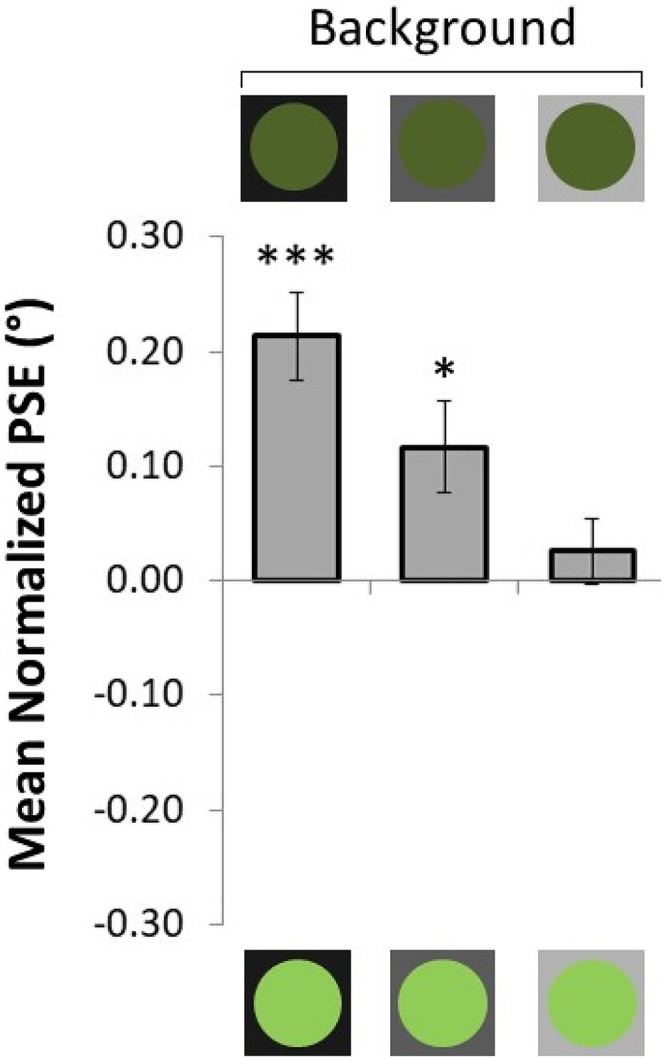
Results of Experiment 3. Here, the mean normalized PSE is plotted for each type of the dresses used as a function of the background luminance. A positive normalized PSE signifies that the dress above the *x*-axis is perceived as thinner than the dress below the *x*-axis, and vice versa. The error bars represent *SEM*. * *p* < .05, *** *p* < .001.

Abbreviations: PSE = point of subjective equality; *SEM *= standard error of the mean.

Figure 7 presents the results of Experiment 3. Darker dresses were perceived as thinner for all levels of background luminance, as the mean normalized PSE was significantly different from zero (average PSE across all conditions = .12°, *t*_18_ = 4.65, *p* = .04, *d* = 1.07, 1 − *β* = 0.99). The thinning effect was non-significant in the light background (PSE = 0.03°, *t*_18_ = 0.90, *p* = .38, *d* = 0.21, 1 − *β* = 0.13), but significant in the average (PSE = 0.12°, *t*_18_ = 2.91, *p* = .01, *d* = 0.67, 1 − *β* = 0.79) and dark (PSE = 0.21°, *t*_18_ = 5.54, *p* < .001, *d* = 1.27, 1 − *β* = 0.99) backgrounds. These results directly reject the hypothesis that the thinning effect obtained for darker dresses was due to their higher contrast with the light-grey background. If that was the case, we would expect the dark green dress in the first bar of Figure 7 to have a reduced thinning effect, since it was viewed on a low-contrast dark background. However, the background luminance seemed to have played an enhancing role on the overall thinning effect across all conditions.

To test this effect, we run a repeated measures analysis of variance. Indeed, the main effect of background absolute luminance was significant (*F*_(2,36)_ = 9.04, *p* = .001, $\eta_{p}^{2}=0.33$, 1 − *β* = 0.96), and post hoc pairwise comparisons revealed that the only significant difference was between the light and dark backgrounds (mean PSE difference = 0.18°, *p* = .001).

Although, for the light background condition we failed to replicate the thinning effect reported in Experiment 2 (*p* = .049), the overall results were in the same direction and the magnitude of the effect was similar to Experiment 2 (PSE Experiment 2 = .05° vs. PSE Experiment 3 = .03°). In general, the results corroborated our previous finding that darker stimuli are perceived as thinner. On top of that, the contrast between the stimuli and the background luminance did not significantly affect the thinning effect of darker dresses. Instead, the absolute luminance of the background seemed to have contributed to the overall effect, since darker dresses were perceived even thinner in darker backgrounds.

## Discussion

In the present study, we focused on the perceptual effect of horizontal stripes in clothes. Unlike most previous studies, we compared the effect of horizontal stripes against non-striped stimuli, that is, against a neutral condition. In Experiment 1, we found that participants perceived a striped dress (with light-green and black stripes) to be significantly thinner than an identical non-striped (light-green) version of the same dress, but not when compared to a non-striped black version. Since the thinning effect was significant only when the striped dress was compared to the light-green dress, we hypothesized that luminance might moderate the thinning effect. In Experiment 2, we dissociated the stripes and luminance effects by including two extra stimuli. In this way, it was possible to measure the effect of horizontal stripes and luminance on the perceived body width independently, as well as their aggregating effect. The results showed that, first, when the effect of luminance was controlled for, horizontal stripes had a thinning effect against non-striped stimuli. Second, when the effect of horizontal stripes was controlled for, stimuli with lower luminance had a thinning effect against stimuli with higher luminance, either when both stimuli were striped or non-striped. Third, when both stripes and luminance were taken together, the effect was even larger.

Although it seems that the stripes effect holds when the luminance of the stimuli and the background are held constant, the luminance of the background—against which the stimuli were placed—might have affected the thinning effect of darker stimuli. This is because, in the luminance effect, the darker (vs. lighter) stimuli were further away from the luminance of the background and this luminance difference might have affected size perception. In Experiment 3, we manipulated the background luminance (i.e., creating light, average, and dark backgrounds) to investigate whether the luminance effect of Experiment 2 was due to the increased contrast between the dark dresses and the light-grey background. The results showed that the thinning effect of dark dresses could not be attributed to stimuli-background contrast. Instead, we discovered that when the luminance of the general background is darker, the thinning effect becomes more pronounced.

The thinning effect of low luminance is in line with the irradiation illusion (Helmholtz, 1962), according to which a bright area seems larger than a darker area of similar size (Long & Murtagh, 1984; Oyama & Nanri, 1960), an effect attributable to the non-linear visual processing of dark and light both on retinal (Valeton & van Norren, 1983; Westheimer, 2008) and cortical level (Kremkow et al., 2014). On the cortical level for instance, Kremkow et al. (2014) showed that the ON and OFF neurons operate in dissimilar ways in our visual system, with the OFF neurons responding linearly to external stimuli, and the ON neurons responding in a curvilinear way (overresponding even to limited external stimulation). The practical implication is that dark stimuli are perceived as smaller than bright, not because the size of dark stimuli is underestimated, but rather because the size of bright stimuli is overestimated. As a consequence, the darker dresses in Experiments 2 and 3 were perceived as thinner presumably because participants might have overestimated the size of the brighter dresses.

So far only one study has tested the perceptual effect of horizontal stripes against non-striped stimuli. Thompson and Mikellidou (2011; Experiment 3) found that a cylinder with horizontal stripes was perceived as similar in size (neither wider nor thinner) to an identical non-striped cylinder. This finding is at odds with the results of the present experiments. Nevertheless, the present results suggest that the thinning effect of horizontal stripes seems to be consistent and strong (in terms of effect size and observed statistical power). They also suggest that the thinning effect is further decomposed into two separate effects, that is, both the orientation and the luminance of the stripes affected the perceived size of the dress, and the thinning effect of luminance was further amplified as the luminance of the background decreased.

Our results also extend and partially explain the Helmholtz illusion. In the original paradigm, a square with horizontal stripes is compared to a similar square with vertical stripes. In that context, the thinning effect of horizontal stripes cannot be attributed to luminance, since both stimuli have the same luminance regardless of stripe orientation. It can be attributed, however, to the thinning effect of horizontal stripes, as Experiment 2 showed. Whether vertical stripes also contribute to the overall effect or not, remains an open empirical question.

### Future Research

The present results open two main venues for future research. The first is to further probe the boundary conditions that might account for the effect. In the present study we tested the effect of horizontal stripes, luminance, and contrast but the list of confounding variables may be longer. Some variables that might account for the effect include the orientation of the stripes (e.g., test the effect of vertical stripes against a neutral condition), their spatial frequency (the thickness of the stripes), or color. Moreover, previous studies reported that the body size moderates the effect, with bigger body types decreasing (Ashida et al., 2013) or even reversing the thinning effect of horizontal stripes (Imai, 1982). Future studies can test the effect in different body sizes and body shapes (e.g., hourglass, spoon, rectangle).

The second line of research calls for an interdisciplinary approach between the fields of social cognition (participants’ previous beliefs) and psychophysics (participants’ perception). It is striking that, although the results of the present experiments support the thinning effect of horizontal stripes, this finding clashes with participants’ beliefs and leads to a paradox: during the behavioral task in the lab participants perceived horizontal stripes in clothes as thinner, but in self-reports they stated the opposite.^3^ This inconsistency was also described by Ridgway et al. (2017) where they scanned the body of 15 women and their 3D avatars were presented on a computer. The avatars were dressed in horizontal stripes (and seven more optical illusions) and participants were asked to evaluate them. A thematic analysis (a qualitative in-depth interview method) revealed that a number of participants swayed away from their original negative attitude towards horizontal stripes once they saw themselves in the horizontally striped dress. Although the results of that study were based on qualitative analysis and did not provide any conclusive evidence, the discrepancy between previous beliefs and behavioral responses regarding the effect of horizontal stripes in clothes still remains.

## Conclusion

The story of stripes in clothes is old and inconsistent. From a symbol of sorcery in medieval times to the sartorial choice of inmates in previous decades, stripes today are a symbol of style and fashion (Pastoureau, 2001). Contrary to the common belief that stripes make someone look wider, across three laboratory experiments we found that a dress with horizontal stripes is perceived as significantly thinner than a similar non-striped dress. We further found that the thinning effect of horizontal stripes can be further decomposed into two separate effects: horizontal stripes and lower luminance exert a thinning effect, and that these two effects seem to sum together, making the thinning effect even stronger. Regarding the luminance effect, the results could not be explained by the luminance contrast between stimuli and background; instead, the thinning effect of darker stimuli was further amplified by background luminance, such that darker stimuli were perceived even thinner as the luminance of the background decreased. Although we cannot offer a direct explanation for this effect, it does seem to be in accordance with the general direction of the irradiation illusion, in which brighter surfaces tend to appear larger than darker surfaces.

## Notes

1. In Experiments 2 and 3 we do not report the distribution of responses, but they were also normally distributed—very similar to Figure 3.

2. *d* refers to Cohen's *d*; 1 − *β* refers to the observed statistical power.

3. One week after Experiment 2 we asked participants whether they believed that horizontal stripes make someone look fatter, thinner, or neither fatter nor thinner. Of the 31 participants who responded, 58% stated that horizontal stripes have a fattening effect, 26% stated that horizontal stripes have a thinning effect, and 16% stated that horizontal stripes have neither effect.

## References

[bibr1-03010066211038158] Alais D. Orchard-Mills E. Van der Burg E. (2014). Auditory frequency perception adapts rapidly to the immediate past. Attention, Perception, and Psychophysics, 77, 896–906. 10.3758/s13414-014-0812-2 25522831

[bibr2-03010066211038158] Ashida H. Kuraguchi K. Miyoshi K. (2013). Helmholtz illusion makes you look fit only when you are already fit, but not for everyone. i-Perception, 4, 347–351. 10.1068/i0595rep 24349693 PMC3859551

[bibr3-03010066211038158] Bowen H. J. Spaniol J. Patel R. Voss A. (2016). A diffusion model analysis of decision biases affecting delayed recognition of emotional stimuli. PloS one, 11, Article e0146769. 10.1371/journal.pone.0146769 PMC4718681 26784108

[bibr4-03010066211038158] Bradshaw M. F. Watt S. J. (2002). A dissociation of perception and action in normal human observers: The effect of temporal-delay. Neuropsychologia, 40, 1766–1778. 10.1016/S0028-3932(02)00039-8 12062888

[bibr5-03010066211038158] Economou E. Zdravkovic S. Gilchrist A. (2007). Anchoring versus spatial filtering accounts of simultaneous lightness contrast. Journal of Vision, 7, 1–15. 10.1167/7.12.2 17997644

[bibr6-03010066211038158] Imai S. (1982). Tateshima yokoshima no nazo (in Japanese). Psychology, 29, 12. Tokyo: Saiensu-sha.

[bibr7-03010066211038158] Helmholtz H. (1962). Treatise on physiological optics (J. P. C. Southall, Trans.; Vol. 3). Dover. (Original work published in 1867).

[bibr8-03010066211038158] Holm S. (1979). A simple sequentially rejective multiple test procedure. Scandinavian Journal of Statistics, 6, 65–70.

[bibr9-03010066211038158] Kremkow J. Jin J. Komban S. J. Wang Y. Lashgari R. Li X. Jansen M. Zaidi Q. Alonso J. M. (2014). Neuronal nonlinearity explains greater visual spatial resolution for darks than lights. Proceedings of the National Academy of Sciences, 111, 3170–3175. 10.1073/pnas.1310442111 PMC3939872 24516130

[bibr10-03010066211038158] Long G. M. Murtagh M. P. (1984). Task and size effects in the Oppel-Kundt and irradiation illusions. The Journal of General Psychology, 111, 229–240. 10.1080/00221309.1984.9921112 6512517

[bibr11-03010066211038158] Mathôt S. Schreij D. Theeuwes J. (2012). Opensesame: An open-source, graphical experiment builder for the social sciences. Behavior Research Methods, 44, 314–324. 10.3758/s13428-011-0168-7 22083660 PMC3356517

[bibr12-03010066211038158] Oyama T. Nanri R. (1960). The effects of hue and brightness on the size perception. Japanese Psychological Research, 2, 13–20. 10.4992/psycholres1954.2.13

[bibr13-03010066211038158] Pastoureau M. (2001). The Devil's Cloth: A history of stripes and striped fabric (J. Cladding, Trans.). Columbia University Press. 10.1080/17518350.2003.11428637

[bibr14-03010066211038158] Ridgway J. L. Parsons J. Sohn M. (2017). Creating a more ideal self through the use of clothing: An exploratory study of women’s perceptions of optical illusion garments. Clothing and Textiles Research Journal, 35, 111–127. 10.1177/0887302X16678335

[bibr15-03010066211038158] Simons D. J. Chabris C. F. (1999). Gorillas in our midst: Sustained inattentional blindness for dynamic events. Perception, 28, 1059–1074. 10.1068/p281059 10694957

[bibr16-03010066211038158] Swami V. Harris A. S. (2012). The effects of striped clothing on perceptions of body size. Social Behavior and Personality, 40, 1239–1244. 10.2224/sbp.2012.40.8.1239

[bibr17-03010066211038158] Swami V. Salem N. Furnham A. Tovée M. (2008). Initial examination of the validity and reliability of the female photographic figure rating scale for body image assessment. Personality and Individual Differences, 44, 1752–1761. https://doi.org/bnm2sz

[bibr23-03010066211038158] The GIMP Development Team. (2019). GIMP. https://www.gimp.org

[bibr18-03010066211038158] Thompson P. Mikellidou K. (2011). Applying Helmholtz illusion to fashion: Horizontal stripes won’t make you look fatter. i-Perception, 2, 69–76. 10.1068/i0405 23145226 PMC3485773

[bibr19-03010066211038158] Valeton J. M. van Norren D. (1983). Light adaptation of primate cones: An analysis based on extracellular data. Vision Research, 23, 1539–1547. 10.1016/0042-6989(83)90167-0 6666056

[bibr20-03010066211038158] Van der Burg E. Olivers C. N. L. Bronkhorst A. W. Theeuwes J. (2008). Audiovisual events capture attention: Evidence from temporal order judgments. Journal of Vision, 8, 1–10. 10.1167/8.5.2 18842073

[bibr21-03010066211038158] Van Eijk R. L. J. Kohlrausch A. Juola J. F. Van de Par S. (2008). Audiovisual synchrony and temporal order judgments: Effects of experimental method and stimulus type. Perception and Psychophysics, 70, 955–968. 10.3758/PP.70.6.955 18717383

[bibr22-03010066211038158] Westheimer G. (2008). Illusions in the spatial sense of the eye: Geometrical-optical illusions and the neural representation of space. Vision Research, 48, 2128–2142. 10.1016/j.visres.2008.05.016 18606433

